# Production and Partial Purification of Alpha Amylase from *Bacillus subtilis* (MTCC 121) Using Solid State Fermentation

**DOI:** 10.1155/2014/568141

**Published:** 2014-01-12

**Authors:** Dibyangana Raul, Tania Biswas, Suchita Mukhopadhyay, Shrayan Kumar Das, Suvroma Gupta

**Affiliations:** Department of Biotechnology, Haldia Institute of Technology, ICARE Complex, Purba Medinipur 721657, India

## Abstract

Amylase is an enzyme that catalyzes the breakdown of starch into sugars and plays a pivotal role in a variety of areas like use as digestives, for the production of ethanol and high fructose corn syrup, detergents, desiring of textiles, modified starches, hydrolysis of oil-field drilling fluids, and paper recycling. In the present work, solid state fermentation (SSF) for **α**-amylase production has been used in lieu of submerged fermentation (SmF) due to its simple technique, low capital investment, lower levels of catabolite repression, and better product recovery. *Bacillus subtilis* has been well known as producer of alpha amylase and was tested using solid state fermentation for 48 hours at 37°C with wheat bran as substrate. Comparison between different fermentation hours demonstrated high yield of alpha amylase after 48 hours. This alpha amylase has optimum pH and temperature at 7.1 and 40°C, respectively. With the goal to purify alpha amylase, 30–70% (NH_4_)_2_SO_4_ cut concentrated the amylase activity threefold with respect to crude fermented extract. This was verified in quantitative DNS assay method as well as in zymogram gel profile. The exact molecular weight of the amylase is yet to be determined with the aid of other protein purification techniques.

## 1. Introduction

Amylase catalyses the breakdown of starch into sugars. *α*-Amylase can breakdown long-chain carbohydrates, ultimately yielding maltose from amylose, or maltose, glucose, and “limit dextrin” from amylopectin. Amylases are produced by a wide spectrum of organisms, although each source produces biochemical phenotypes that significantly differ in parameters like pH and temperature optima as well as metal ion requirements [1]. Till date, two major classes of amylases have been identified in microorganisms, namely, *α*-amylase and glucoamylase. *α*-Amylases (endo-1,4-a-D-glucan glucohydrolase) are extracellular enzymes that randomly cleave the 1,4-a-D-glucosidic linkages between adjacent glucose units in the linear amylase chain. Glucoamylase (exo-1,4-a-D-glucan glucanohydrolase) hydrolyzes single glucose units from the nonreducing ends of amylose and amylopectin in a stepwise manner [1, 2]. These are calcium metalloenzymes, which are completely unable to function in the absence of calcium. Calcium stabilizes the interface between the central A domain (291 residues) with (*β*/*α*)_8_ barrel structure and the more variable B domain (104 to 206 residues) [3–7].

Because of its multifarious application amylase attracts attention of researchers since decades after its first isolation and identification in the year 1894 from a fungal source that was used as additives in pharmaceutical digestive formulations [8]. Since the onset of its discovery the research continues till date involving microorganisms as a potential source of amylase. Microorganisms are chosen preferentially for amylase production due to the relative ease of handling, availability, favorable growth conditions, and cheap nutrient requirement compared to other producers like plant and animal. Amylase occupies a major share (around 25%) of total world enzyme market owing to its high demand eliminating chemical hydrolysis of starch in the starch liquefaction process [9, 10]. It has been utilized also in textile, food, brewing, and paper pulp industries [11–13].

SmF has been a traditional way for production of industrially important enzymes since long past due to multiple facilities like better control over environmental factors namely, pH, temperature, aeration, and moisture level. Cultures reported to be utilized for amylase production using SmF belong to a variety of *Bacillus* species like *Bacillus *sp. PN5, *Bacillus subtilis *JS-2004, *Bacillus *sp. IMD 435, *Bacillus *sp. I-3, *Bacillus caldolyticus *DSM405, *Bacillus licheniformis *GCBU-8, and so forth [14–19]. However, SSF replaces SmF as it mimics the natural habitat of microorganisms. SSF is a better choice over SmF due to its simplicity, low capital investment, lower energy requirement, less water output, and lack of foam built up [20–22].

With the advent of biotechnological innovations especially in the area of fermentation technology and enzyme production, SSF with agro wastes like WB, RB, COC, and GOC has replaced the high cost media generally used in submerged fermentation for alpha amylase preparation.* Bacillus *species are frequently used for **α**-amylase production with the aid of SSF using wheat bran [23–29].

Keeping in mind that raw, partially purified amylase, has been used in digestives, in the present work, we have partially purified the *α*-amylase extracted from *Bacillus subtilis*. Knowing that solid state fermentation (SSF) is much cost effective and efficient than submerged fermentation (SmF), we have used wheat bran as substrates for the production of *α*-amylase. The specific activity of the crude and purified enzyme was determined using DNS assay and Lowry method [30, 31]. The pH and temperature optimum of alpha amylase have been determined. Amylase from the fermented extract has been partially purified using ammonium sulphate cut and verified in native PAGE as well as in zymogram.

## 2. Materials and Methods

### 2.1. Preparation of Inoculum for Solid State Fermentation

1.5 mL inoculum has been added to 50 mL LB media. After inoculating the media with the culture, the media was kept at 37°C for 48 hr and the O.D was checked (O.D_600_ = 0.450). Inoculum having OD equivalent to O.D_600_ = 0.450 was added to 4 gram of autoclaved wheat bran (WB) available from local market for SSF and kept at 37°C temperature for 48 hours fermentation period.

### 2.2. Enzyme Extraction

After 48 hours of fermentation the fermented media were taken out and soaked in 20 mM phosphate buffer (pH = 7.0) for 30 minutes at 4°C in a rotary shaker. It was centrifuged at 8000 rpm for 15 min at 4°C. After this the supernatant has been collected which is enzyme extract.

### 2.3. Amylase Assay

Alpha amylase activity of the extract was measured by DNS method [30]. In brief the reaction mixture containing 1% soluble starch, 20 mM phosphate buffer (pH = 7), and fermented extract was taken and incubates at 37°C for 20 minutes followed by the addition of 3,5-dinitrosalicylic acid (DNS). The amount of the reducing sugar liberated during assay was estimated by measuring color development at 540 nm by UV-VIS spectrophotometer. 1U of amylase activity is defined as the amount of enzyme that liberated micromole of maltose per minute under standard assay condition.

### 2.4. Protein Estimation

The protein content of the extract was determined following Lowry's method [31].

## 3. Purification of Alpha Amylase Using Ammonium Sulphate Precipitation

For (0–30) % cut, 10 mL of enzyme extract was taken in falcon and (268 mg × 10 i.e., 2.68 gm.) of ammonium sulphate is added slowly in the extract. The mixture was stirred thoroughly for 30 minutes. Then the solution was centrifuged, the pellet was taken out, and the supernatant was kept. After that the pellet was resuspended in 2 mL of 20 mM phosphate buffer.

For (30–70) % cut, the retrieved supernatant was taken and 2.56 gm. of ammonium sulphate was added to it. The previous steps for dialysis were repeated again.

### 3.1. Native PAGE and Zymogram

The composition of 10 mL of resolving gel includes 4 mL dH_2_O, 2.50 mL 1.5 M Tris, 2.667 mL Bis-Acrylamide, 0.733 mL 1% APS, and 5 *μ*L TEMED. After sometime, when the resolving gel was set, the stacking gel was poured and allowed to get settled. The composition of 10 mL of stacking gel consisted of 5.70 mL dH_2_O, 2.50 mL 0.5 M Tris, 1.0 mL Bis-Acrylamide, and 0.70 mL 1% APS, 10 *μ*L TEMED.

Now the gel was developed for zymogram by dipping in 1% starch solution for 5 minutes followed by addition of few drops of iodine to it. 1% starch solution was prepared with 1gm of starch in 100 mL of 20 mM phosphate buffer. In order to prepare iodine solution, 10 gm of KI crystals was dissolved in 100 mL distilled water and 5 gm of iodine was added to it.

## 4. Results and Discussion

*Bacillus* species are considered to be the most important sources of *α*-amylase and have been used frequently for enzyme production using SSF.

### 4.1. Solid State Fermentation for Production of *α*-Amylase

After 48 hours of solid state fermentation at 37°C using *Bacillus subtilis,* the production of *α*-amylase was detected by determining enzymatic activity using DNS method at 40°C. The specific activity of amylase was 13.14 (*μ*mol/mg/min) at 40°C (Table 1).

### 4.2. Determination of Amylase Activity at Different Temperature and pH

**Figure 1:**
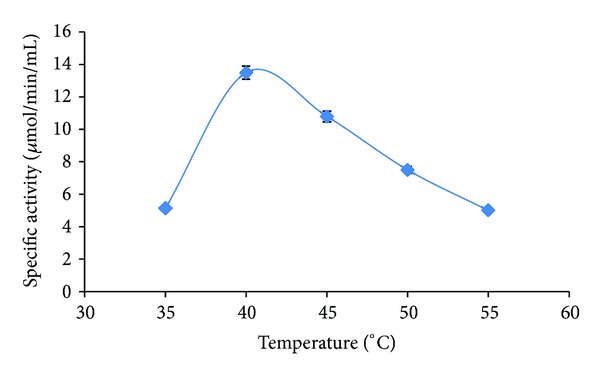
Effect of temperature on specific activity of *α*-amylase.

Temperature and pH have profound effect on enzymatic activity. 48 hours of fermented extract exhibited optimum temperature for amylase as observed from Figure 1 which is around 40°C. The specific activity of amylase was 13.14 *μ*mol/mg/min at 40°C.

**Figure 2:**
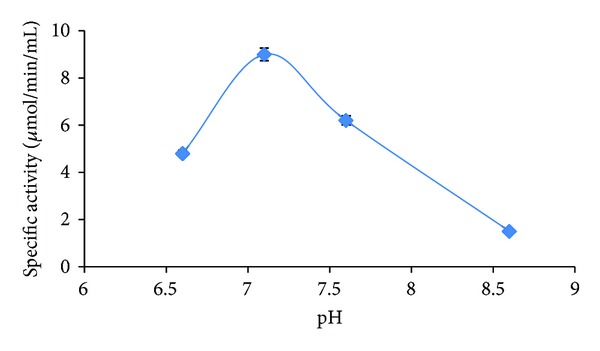
Effect of pH on specific activity of *α*-amylase.

For determination of suitable pH range for enzymatic activity, pH enzyme assay buffers were varied as 6.6, 7.1, 7.6, and 8.6. Maximum enzyme activity was observed at pH 7.1 (8.74 *μ*mol/mg/mL) at 40°C. The use of more alkaline buffer resulted in sharp decline of enzyme activity (Figure 2).

## 5. Partial Purification of ***α***-Amylase

Partial purification of *α*-amylase from crude enzyme extract obtained from solid state fermentation of wheat bran was achieved using (NH_4_)_2_SO_4_ precipitation. The enzyme assay was performed to determine the *α*-amylase activity in ammonium sulphate fractions. Localization of amylase activity was seen in the 30–70% fraction of supernatant obtained from (0–30%) cut compared to the enzyme activity from 0–30% fraction. There is an approximate 3-fold increase in specific activity compared to crude. The specific activity of amylase was 7.73 *μ*mol/min/mg at 40°C in 30–70% whereas 1.47 *μ*mol/min/mg specific activity was found in the other fraction 0–30%.

## 6. Characterisation of ***α***-Amylase in PAGE

**Figure 3:**
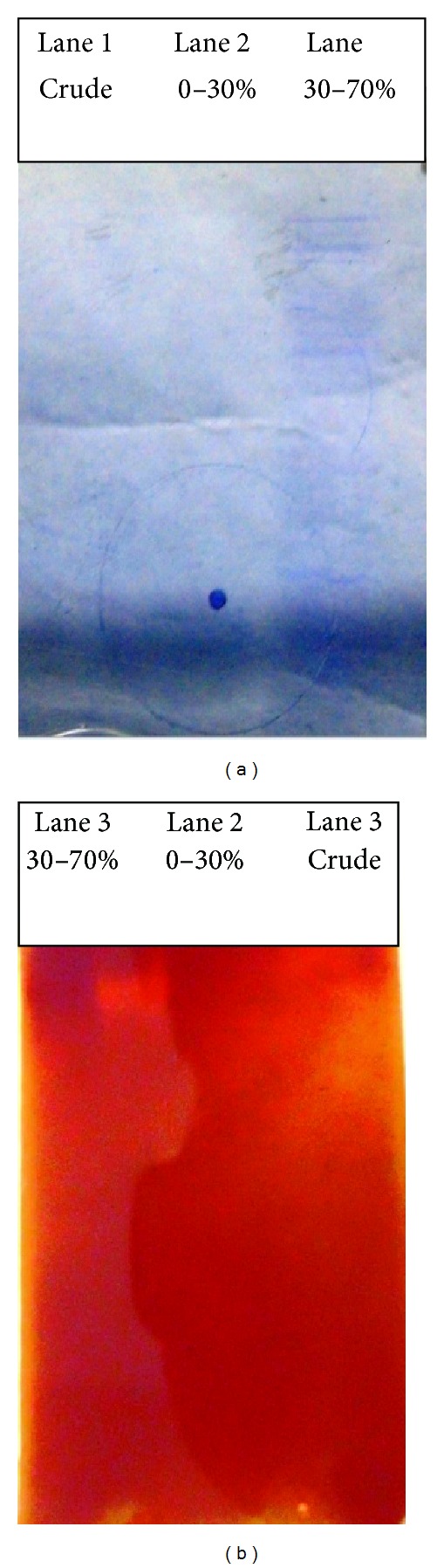
(a) 8% native gel profile; Lane 1: crude extract, Lane 2: 0–30% cut; Lane 3: 30–70% ammonium sulphate cut from SSF. (b) Zymogram for amylase activity. Lane 1: crude extract; Lane 2: 0–30% cut; Lane 3: 30–70% ammonium sulphate cut from SSF.

Alpha amylase obtained from 48 hours of fermented extract was characterized in 8% native gel. The observation was presented in Figure 3; from the gel profile it was evident that, relative to the crude extract, 30–70% (NH_4_)_2_SO_4_ fraction was enriched in protein. This also corroborates with quantitative determination of amylase activity (Table 2). It was observed that 0–30% (NH_4_)_2_SO_4_ fraction had relatively poor amylase activity with respect to its protein content (specific activity = 1.47 *μ*mol/min/mg). This data is validated from Figure 3 where in lane 2 complete disappearance of protein band was in accordance with the minute activity of amylase (specific activity = 1.47 *μ*mol/min/mg).

In order to confirm the presence of amylase in 30–70% (NH_4_)_2_SO_4_ fraction, zymogram for alpha amylase was conducted using the other half of the native gel. The observation was noted in Figure 3(b). In lane 3, amylase activity was detected as a clear band in dark background in presence of starch iodine. Absence of clear band due to amylase activity in lanes 1 and 2 corresponding to crude and 0–30% fraction demonstrated relative purification of amylase in 30–70% fraction. *α*-Amylase activity is due to the conversion of limit dextrin under the influence of an excess of *β*-amylase to products which give red-brown coloration with iodine. Molecular weight of *α*-amylase from *Bacillus* species ranges between 50 and 60 kDa though some exception exists in case of *α*-amylase (molecular weight 31 kDa) isolated from *Bacillus licheniformis* [32–34]. In the present study partial purification of *α*-amylase limits our knowledge to conclude specifically regarding the molecular weight of *α* amylase. Further work is to be performed to delineate molecular mass of the same.

High market demand of amylases with specific applications in the food and pharmaceutical industries necessitates production and partial purification of this valuable enzyme using cheap raw material like wheat bran from SSF. With this goal, the present work is attempted to purify amylase from *Bacillus subtilis that* can be used in number of areas like detergent, textiles, hydrolysis of oil-field drilling fluids, and paper industry.

## 7. Conclusion

Though some preliminary enzymatic parameters like pH optimum and temperature optimum have been determined for alpha amylase from 48 hours of SSF at 37°C, complete purification as well as molecular weight determination is still to be performed. At present, with the goal to purify alpha amylase, partial concentration of enzyme was achieved through 30–70% (NH_4_)_2_SO_4_ cut, as verified from zymogram pattern. Further work for complete purification of alpha amylase would be conducted with the aid of other purification techniques.

## Figures and Tables

**Table 1:** *α*-Amylase specific activity of 48 hours of fermented extract.

Fermentation period	Extract activity (*µ*mol/mL/min)	Protein concentration (mg/mL)	Specific activity (*µ*mol/min/mg)
48 hrs extract	2.243	0.17	13.19

**Table 2:** Partial purification of alpha amylase.

Sample	Activity (mg/mL/min)	Protein concentration (mg/mL)	Specific activity (*µ*mol/min/mg)
Crude	1.56	2	2.27
(0–30%) (NH_4_)_2_SO_4_ cut of crude enzyme	0.208	0.41	1.47
(30–70%) (NH_4_)_2_SO_4_ cut of supernatant obtained from (0–30%) (NH_4_)_2_SO_4_ cut	3.185	1.2	7.72
